# Comparison of two established 2D staging techniques to their appliance in 3D cone beam computer-tomography for dental age estimation

**DOI:** 10.1038/s41598-021-88379-1

**Published:** 2021-04-27

**Authors:** Matthias Zirk, Joachim E. Zoeller, Max-Philipp Lentzen, Laura Bergeest, Johannes Buller, Max Zinser

**Affiliations:** Department for Oral and Craniomaxillofacial and Plastic Surgery, University of Cologne, Kerpener Strasse 62, 50931 Cologne, Germany

**Keywords:** Anatomy, Medical research, Signs and symptoms

## Abstract

For medicolegal purposes, orthodontic or orthognathic treatment various stomatological staging technique for age estimation with appliance of conventional radiographic images have been published. It remains uninvestigated if cone beam computer-tomography delivers comparable staging results to the conventional radiographic stages of third molar analysis. We conducted a retrospective cross-sectional study of 312 patients aged 13–21 years. Dental age estimation staging technique, introduced by Nolla and Demirjian, were applied on the left lower third molar imaged by conventional panoramic radiographs and cone beam computer-tomography. It was investigated if 2D and 3D imaging presented different staging results for dental age estimation. In 21% the Demirjian’s staging differed by a single stage between 2 and 3D images. The greatest congruence (87%) between 2 and 3D images was revealed for stage 7 (G). In contrary, stage 5 (E) presented the lowest level of congruence with 47.4%. The categorization of Nolla revealed divergences in staging for than two categorical variables in Nolla’s stages 3, 4, 5 and 6. In general, the analysis of the data displayed the divergence for Nolla’s stages 4–8. The staging results for 2D and 3D imaging in accordance to the rules of Nolla and Demirjian showed significant differences. Individuals of 18 years may present immature third molars, thus merely an immature third molar cannot reject legal majority. Nolla’s and Demirjian’s 2D and 3D imaging present significantly different staging results.

## Introduction

The estimation of age remains part of active research in forensic science^1^. Various staging techniques have been published before, however, merely some staging technique are considered to have an acceptable scientific basis^1,2^. Moreover, for accurate age estimation of living individuals in legal proceedings a series of clinical and radiological examinations are carried out. The age estimation procedure often includes a physical examination, dental examination with dental status and an X-ray of the dentition and an X-ray examination of the left hand or in addition the clavicles^3^. Thus, stomatological staging techniques remain a key part of age estimation. In particular in a medicolegal context, the development of the third molar aids as an age indicator especially in the adolescence^4^. The third molar’s development is mostly impacted by genetics, whereas environmental factors seem to have a lesser effect on the third molars development^5^. The dental development follows a regular pattern and is radiologically assessable^6^. In comparison to the skeletal development, the dental development is delayed and slower^6^. For radiographic imaging in age estimation, a great variety of 2D studies have been conducted, more recently several 3D studies with focus on the dental pulp have emerged^7,8^. However, there is a need for more retrospective investigation of 3D data to create feasible tools for age determination with 3D imaging^9^.

The two-dimensional radiographs, even if properly executed, are limited in providing an accurate view of a three-dimensional object. Consequently, in modern dentistry cone beam computed tomography (CBCT) have become a useful diagnostic and scientific tool to evaluate the dentition^10,11^. Furthermore, dental imaging is less invasive than diagnostics based on osseous analysis, yet has shown similar or superior accuracy in adults^7^.

In age estimation several schemes have been applied^12^. The Demirjian’s method, introduced in 1973, is a classification of mineralization stages from the first incisor to the second molar of the left mandible^12,13^. Hereby, each tooth is assigned a stage from A (beginning mineralization) to H (apex closed), the stages can be converted into maturity scores. Finally, an age estimation is obtained by the sum of the maturity scores^12,13^. Commonly, the Demirjian’s method is applicable for individuals up to 16 years of age as the evaluation method is confined to the development of the permanent second molar^14^. However, the attainment of 18 years is an important mark in medico-legal purposes, thus earlier studies applied the Demirjian’s method to the third molar for age estimation^15–17^.

The Nolla’s method was first described in 1960 for age estimation^18^. Nowadays, it is one of the least frequently used and tested staging technique, despite its effectiveness^19^. In Nolla’s method each tooth is assigned to a certain stage ranging from stage 0 (the absence of crypt) or stage 1 (presence of a crypt) to stage 10 (the completion of the tooth root’s apical end)^18^. Recently, Nolla’s method has been applied on the third molar in combination with the third molar index (I_3M_) to discriminate adults from minors with high specificity^20^. Further studies demonstrated the applicability of Nolla’s method to the third molar in age estimation as a useful tool^21,22^. In literature, several studies have confirmed the results of Nolla’s method to be no less reliable than further staging techniques such as the more commonly applied Demirjian’s method^21,23,24^.

The age estimation staging technique, introduced by Nolla and Demirjian, are conducted with two-dimensional X-ray technique, e.g. panoramic radiographic images^25^. More data seems necessary to verify the applicability of the staging techniques of Nolla and Demirjian in three-dimensional CBCT scans.

Thus, we investigated if the dental staging techniques of Nolla and Demirjian for conventional 2D panoramic radiographs and 3D cone beam computer-tomography images present different results for adultescents and young adults. Furthermore, we investigated how 3D imaging of the third molar can aid in age estimation with the staging techniques described by Nolla and Demirjian.

## Results

### Comparison of Demirjian’s method in 2D/3D on third lower molar

**Figure 1:**
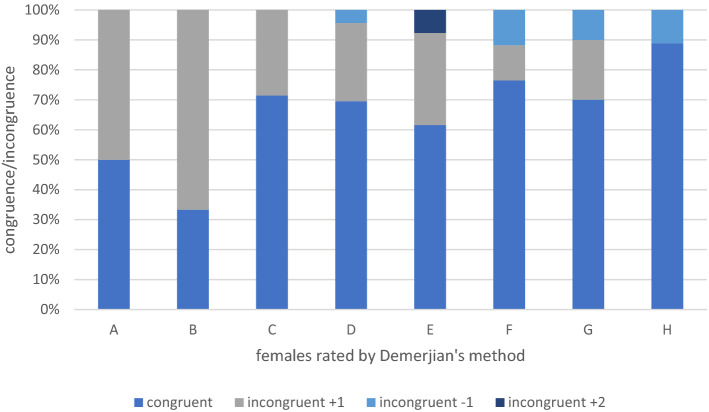
The congruent and incongruent ratings by Demerjian’s method are presented for the female cohort.

In the female cohort 87 panoramic radiographs and CBCT were assessed. Hereby 59 patients presented matching results in the 2D and 3D radiographic diagnostics and therefore were labeled with the same stage. Whereas 22 females were rated differently in the 3D diagnostic compared to their results in the 2D evaluation. The largest divergence was detected in stage 2 (*fusion of cusps*^13^) with 66.7%. Stage 6 (*root length is equal to or greater than the crown height*^13^) presented the second largest divergence between the evaluation of 2D and 3D images with 11.8%. In summary, categorial divergence by a single stage was detected in 5.7% between 2 and 3D images (Table 1). Furthermore, categorial divergence by more than single stage was present in 1.2%. The categorization of Demirjian revealed significantly diverse staging results in comparison of 2D and 3 D images of female patients, p < 0.05 (Fig. 1).

**Table 1:** Patient’s age with Demirjian’s categorical staging.

Stages	Male age—2D	Male age—3D	Female—2D	Female—3D
1 (A)	13.0658 ±		12.8753 ± 0.43843	12.5288 ± 0.44170
2 (B)	13.4493 ± 1.27956	13.5900 ± 1.15830	14.0347 ± 1.81537	12.9352 ± 0.65791
3 (C)	14.1295 ± 1.28633	13.5822 ± 1.53011	13.8614 ± 1.36235	14.6504 ± 2.24675
4 (D)	14.7696 ± 1.96717	14.3353 ± 1.49189	15.9635 ± 1.99192	15.0047 ± 1.60657
5 (E)	16.2643 ± 1.58383	16.3745 ± 1.68727	15.8849 ± 1.20645	16.6166 ± 1.75269
6 (F)	16.8819 ± 1.37914	16.7378 ± 1.49705	16.5679 ± 1.63789	16.1288 ± 1.34381
7 (G)	17.5721 ± 1.11853	17.5238 ± 1.16615	17.2137 ± 1.95771	17.1345 ± 1.46936
8 (H)	18.6712 ± 0.87519	18.7576 ± 0.81567	18.8277 ± 1.35279	19.0997 ± 1.16221

Mean and standard deviation (SD) for gender specific age in respect to the staged variables of 2D and 3D images. For variables 2D and 3D images presented significant correlations p < 0.05. Pearson’s correlation coefficient was calculated as r_2D_ = 0.754 for 2D images and as r_3D_ = 0.767.

**Figure 2:**
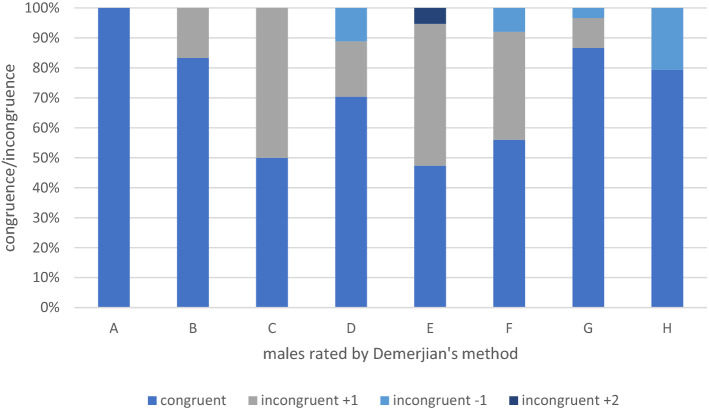
The congruent and incongruent ratings by Demerjian’s method are presented for the male cohort.

In the male cohort 150 panoramic radiographs and CBCT were assessed. Hereby 59 patients presented matching results in the 2D and 3D radiographic diagnostics and therefore were labeled with the same stage. In 31 cases (21%) the categorial staging differed by a single stage between 2 and 3D images. The greatest congruence (87%) between 2 and 3D images was revealed for stage 7 (parallel walls of the root canal and partially open apex^13^. In contrary, stage 5 presented the lowest level of congruence with 47.4%. See Table 1 as well. The categorization of Demirjian revealed significantly diverse staging results in comparison of 2D and 3 D images of male patients, p < 0.05 (Fig. 2).

**Figure 3:**
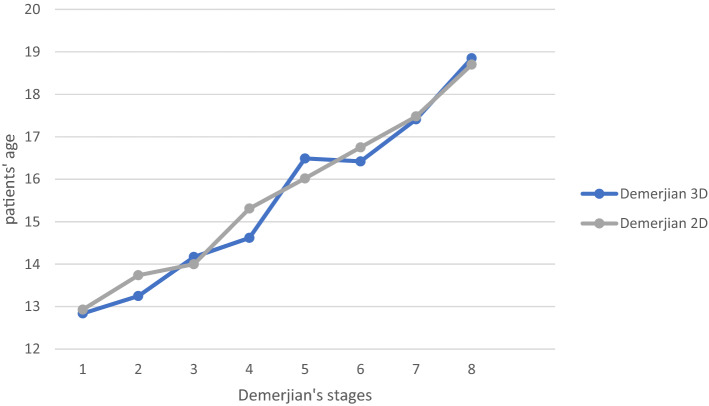
Staging technique of all patients for 2D and 3D analysis. 2D and 3D analysis of Demirjian’s staging technique and patient’s age of both genders is displayed.

**Figure 4:**
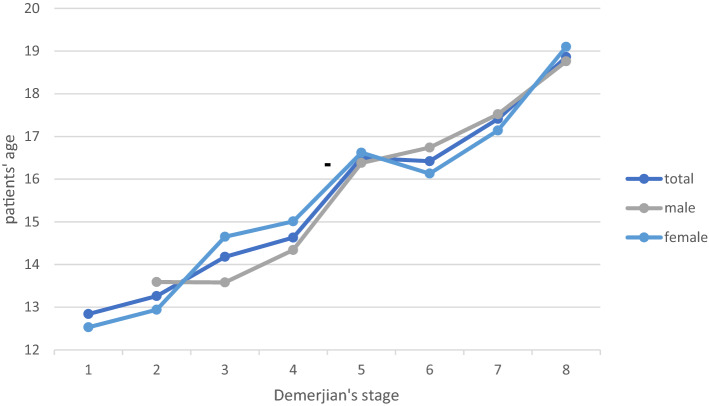
Categorical staging in comparison of both genders in 3D analysis. The slopes of Demerjian’s stages for the female and male cohort in relation to the total cohort are illustrated.

In comparison, 2D and 3D analysis of Demirjian’s categorical variables and patient’s age a linear increase of the 2D-graph is observed, whereas the 3D-graph presents a decrease between stages 5 and 6 (Fig. 3). The none-linear increase of 3D-graph of Demirjian’s categorical variables was particularly observed in the female cohort. In the female cohort a decrease from stage 5 to stage 6 in regard to the female patient’s age was observed (Fig. 4). Notably, the male cohort demonstrates a steady increase between Demirjian’s stage 5 and 6 (Fig. 4). For the male cohort, the graph possesses a slight decrease between Demirjian’s stage 2 and 3 as well as 5 and 6 (Fig. 4). In regard to Demirjian’s method different staging results were obtained from the analysis of 2D and 3D radiographic images.

Furthermore, the evaluation of the 3D images (CBCT) revealed significantly differed Demirjian’s stages between several age groups, the results are displayed in Tables 2 and 3.

**Table 2:** Patient’s age with Nolla’s categorical staging.

Stages	Male age—2D	Male age—3D	Female—2D	Female—3D
1			12.3000 ± 0.11817	12.3836 ±
2	13.4164 ± 1.28781	13.3068 ± 1.40819	13.4952 ± 1.13565	12.7370 ± 0.50932
3	14.3514 ± 1.58491	14.1732 ± 1.37438	14.6712 ± 2.37647	14.7425 ± 1.92391
4	14.1041 ± 1.19309	14.1063 ± 1.67913	13.7282 ± 1.16992	14.7553 ± 2.52858
5	14.6677 ± 2.31089	13.0904 ± 1.25538	15.7089 ± 2.55713	14.5242 ± 1.48895
6	15.0770 ± 1.62411	15.0322 ± 1.36805	15.9464 ± 1.70621	15.4293 ± 1.39593
7	16.5790 ± 1.56545	16.2385 ± 1.88469	16.5817 ± 1.67506	16.7060 ± 1.89137
8	17.1553 ± 1.32243	17.1993 ± 1.34244	16.5365 ± 1.30545	16.0858 ± 1.27075
9	17.5721 ± 1.11853	16.93 ± 1.246	17.2137 ± 1.95771	16.58 ± 1.505
10	18.6712 ± 0.87519	18.27 ± 0.785	18.22 ± 1.394	18.60 ± 1.174

Intra- and inter-examiner reliability in ICC value were calculated as 0.98 (p ≤ 0.05) for Demirjian’s method and 0.96 for Nolla’s method (p ≤ 0.05).

**Table 3:** Age group with significant different presentation of Demirjian’s stages.

B ≠ H	C ≠ H	D ≠ H	E ≠ H
	C ≠ G	D ≠ G	E ≠ G
	C ≠ F		E ≠ F

### Comparison of Nolla’s method in 2D/3D on third lower molar

**Figure 5:**
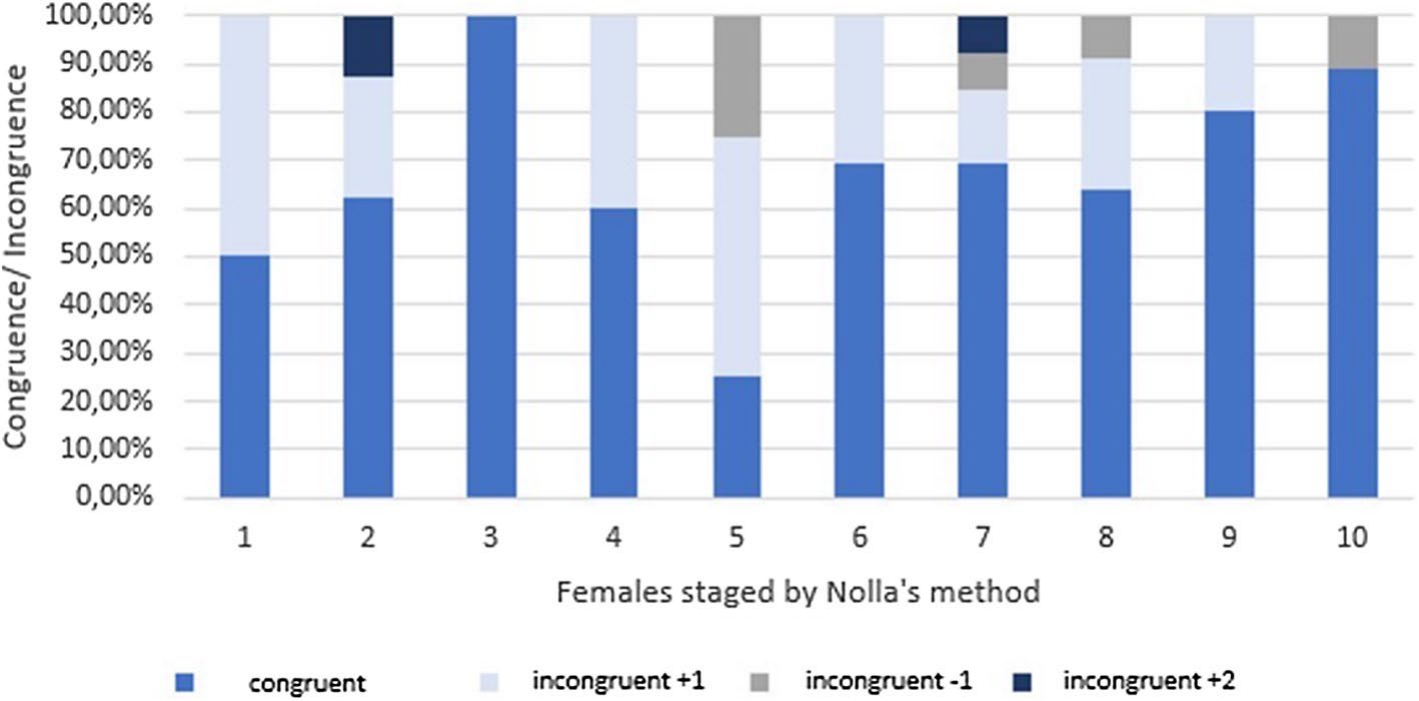
The congruent and incongruent ratings by Nolla’s method are presented for the female cohort.

In the female cohort 89 panoramic radiographs and CBCT were assessed. Hereby 61 patients presented matching results in the 2D and 3D radiographic diagnostics and therefore were labeled with the same stage (Table 2). Whereas 25 females (28%) were rated differently in the 3D diagnostic compared to their results in the 2D evaluation. The greatest divergence was detected in stage 6 of Nolla’s categorical staging^18^. In stage 6 our investigation found 7 patients (~ 8%) differently rated by a single stage (18%). Results are displayed in detail in Fig. 5. Nolla’s stage 3 was equally confirmed for females in analysis of 2D and 3D images^18^.

**Figure 6:**
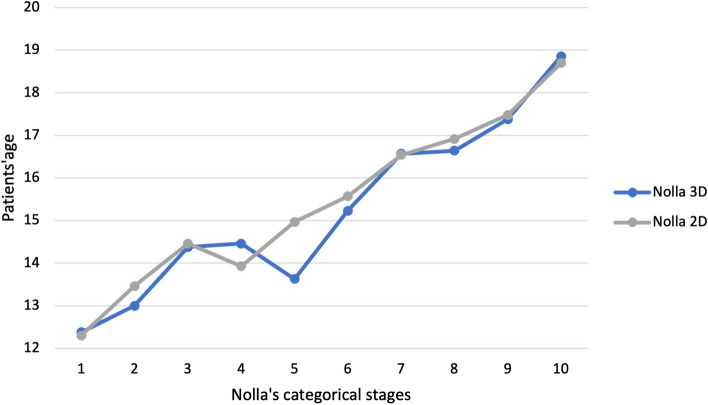
Nolla’s Categorical staging in comparison of both genders in 3D analysis. The slopes of Nolla’s stages for the female and male cohort in relation to the total cohort are illustrated.

**Figure 7:**
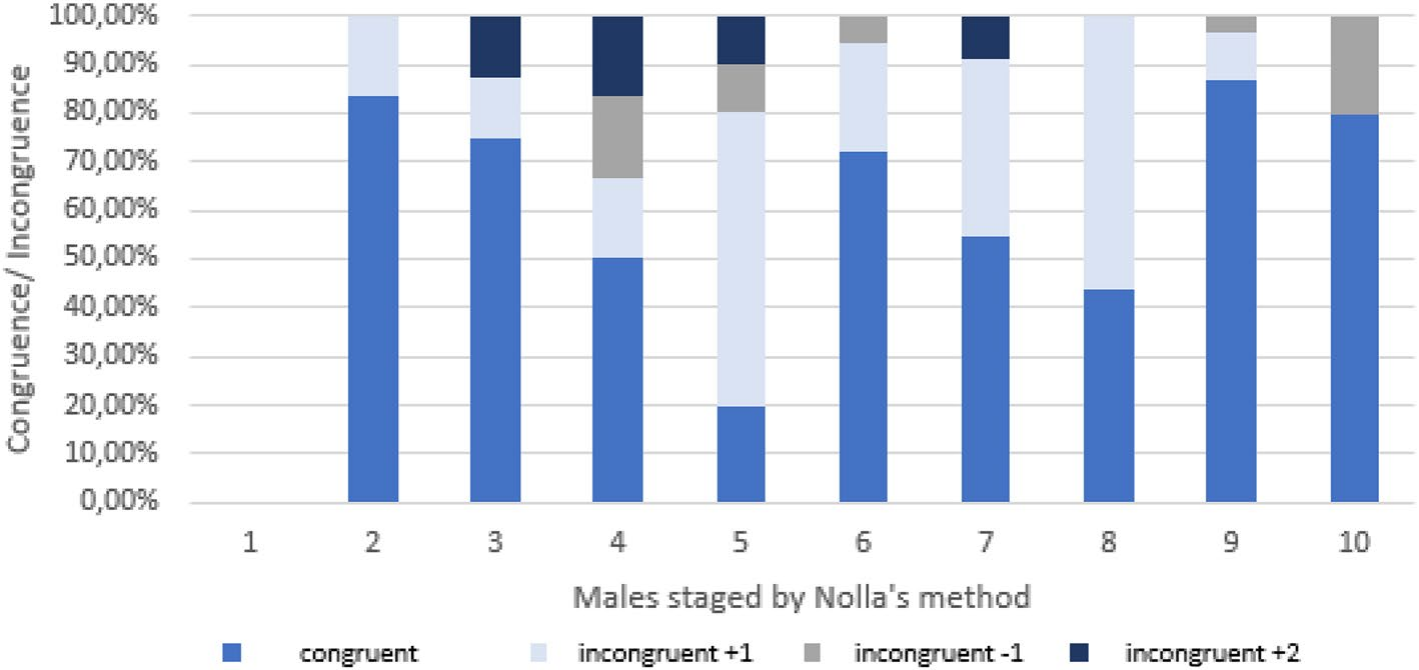
The congruent and incongruent ratings by Nolla’s method are presented for the male cohort.

**Figure 8:**
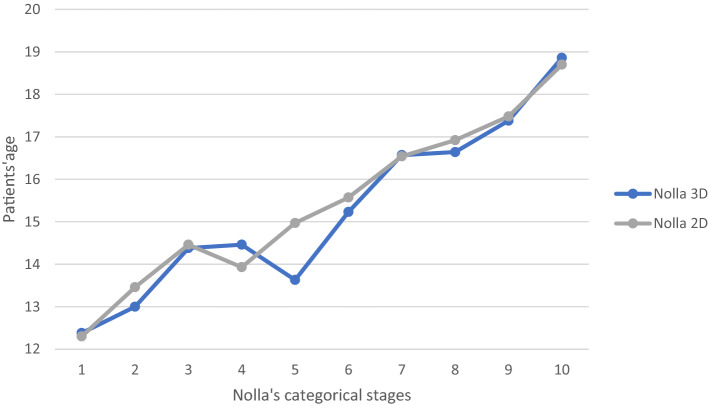
Nolla’s Categorical staging of all patients for 2D and 3D analysis. 2D and 3D analysis of Nolla’s staging technique and patient’s age of both genders is displayed.

In the male cohort 150 panoramic radiographs and CBCT were assessed. Hereby 102 patients presented matching results in the 2D and 3D radiographic diagnostics and therefore were labeled with the same stage. The categorical staging according to the rules set by Nolla^18^ revealed divergences in staging for more up to 2 categorical variables in Nolla’s stages 3, 4, 5 and 6. In general, the analysis of the data displayed the divergence for Nolla’s stages 4–8 (Fig. 6). The categorization of Nolla revealed significantly diverse staging results in comparison of 2D and 3 D images of male patients, p < 0.05 (Fig. 7). Likewise, significant differences were documented in 2D versus 3D images (Fig. 8). Age group differences for Nolla’s stage are displayed in Table 4.

**Table 4:** Age group with significant different presentation of Nolla’s stages.

2 ≠ 10
2 ≠ 8

## Discussion

In this study, we investigated two established staging techniques for age estimation and applied their rules on the third molar. The third molar was chosen because of its individual development in adolescence and young adulthood^26,27^. In age estimation various classification methods for the cycles of dental formation have been tested^27^. Hereby, Demirjian’s method is the most commonly investigated, whereas fewer studies on Nolla’s method have been published^27^. In general, the majority of studies evaluate dental age on the basis of conventional 2D panoramic images, these images may partially distorted by overlapping dental structures^28^. Hence, an investigation of the applicability of established age estimation staging techniques in 3D imaging seems reasonable.

Initially, the rules set by Nolla et al. and Demirjian et al. were established for age estimation of children based on the development of the permanent teeth^18,29,30^. However, the accuracy of dental age estimation based on the development of the permanent teeth decreases in juveniles and adolescents with the exception of the analysis of third molars^29,31^. For third molar analysis, the cone beam computer-tomography allows an accurate diagnostic imaging through its cross-sectional views and contains more detailed information in comparison to the conventional panoramic imaging^32^.

For Demirjian’s method, an overestimation of age is described in previous studies as well as an underestimation of age in comparison to the patient’s chronological age^33^. In our study, we detected an incongruence between 2 and 3D categorical staging in accordance to Demirjian’s rules. In general, the higher stages above 6 (F—the root length is equal or greater to the crown’s height) show a greater congruence of the staging categories (Figs. 3, 4). In the higher categorical stages of Demirjian method, 3 D analysis revealed no clear tendency of neither an overestimation nor an underestimation in our cohort. The original proposal of Demirjian’s dental maturity were derived from the French–Canadian population, however up to this day, their applicability in various populations remains a point of debate^13,30,34^. In assessment of the dentition, especially for dental root morphology, CBCT images possess better sensitivity and specify compared to 2D radiographs^35^. Thus, the accuracy of Demirjian’s categorical staging should be improved by a CBCT based estimation. In our study, we observed a positive linear slope of Demirjian 2D data in regard to the categorical staging. Notably, for the categorical stages 5 (E) and 6 (F) of our study’s cohort, the Demirjian 3 D data’s slope possesses a slight decrease before its incline to categorical stage 7 (G). Thus, this phenomenon might indicate that age-estimation based on a three-dimensional view may detect advanced Demirjian stages in adolescence (15–16 years of age). In contrary, lower Demirjian stage were noted earlier adolescence (13–14 years of age), see Fig. 1. Yet, a larger cohort is needed to definitely verify this observation. Notably, in consideration of the third molar variability in its development, eruption and anatomy the original Demirjian's method excluded wisdom teeth^13,36^.

In age estimation, gender has to be considered. In comparison of males and females in the 3D analysis, males reached Demirjian stages 3 (C) and 4 (D) earlier in the earlier adolescence (13–14 years of age). In particular, females presented the Demirjian stages 6 (F) and 7 (G) earlier in adolescence and late adolescence. To some extent, this gender difference may be attributed to faster biological and dental maturation of females which results in a higher dental age in comparison to females’ chronological age^37^. However, biological variation found in boys have been reported to be larger than the variation found in girls^38^ which can explain the difference illustrated in our data (Figs. 2, 4).

Furthermore, we included Nolla’s staging system to this study. Despite the less frequent appliance of Nolla’s method in comparison to Demirjian’s method, Nolla’s method has proven itself to be a reliable tool for age estimation^24,39^.

In consideration of our data, 2D and 3D imaging presented Nolla’s stage 7 for adolescence at the age of 16 years with great congruence, Figs. 3 and 4. On the other hand, the stage 6 was detected earlier in the 3D CBCT-based age estimation. In literature, the calcification of the third molar’s crown (Nolla stage 6, Demirjian’s stage D) is documented with a great variation among different ethnic populations^22^. Thus, our cohort’s data may reflect the heterogenic European population resided in North Rhine-Westphalia, Germany.

Moreover, three dimensional CBCT images confirmed results from an earlier study on third molars that males reach higher stage of Nolla’s classification before females^40^. Likewise, in our study’s cohort males reached earlier Nolla’s stages 3–7 earlier than females. In addition, CBCT images reveal more advanced stages at an earlier age than the conventional panoramic images, Figs. 3 and 4. This observation verifies earlier reports of under-estimation associated to the Nolla’s method applied for conventional panoramic images is consistent with the findings of other investigators^19,41^.

Previous investigations have shown a relatively low inter- and intraobserver disagreement in appliance of Nolla’s method on the second dentition and the third molar^22^. Up to a certain limit, Demirjian’s and Nolla’s staging techniques delivered comparable results. For example, the crown is completed in Demirjian stage 4 (D) and in Nolla stage 6, but the investigation are mainly limited by the cohort’s own ethnicity^22,42^. Therefore, an age estimation method based on the third molar may lack accuracy in estimation of minors^43^. This observation is confirmed by data in our comparisons of 2D and 3D radiographic images. On the contrary and in line with our study’s data, age estimation based on the third molar is a reliable tool in determination of the adult/child transition^43^. Furthermore, age estimation based on the third molar gains a greater level of accuracy if a 3D imaging tool, such as a CBCT, is applied. Still, there is no dividing line with a 100% certainty to adult age. However, a fully mature third molar signifies adult age with a high likelihood, yet a significant proportion of individuals above the age of 18 have immature third molars^44^. Thus, in order to distinguish between adolescence and adulthood, a completely matured third molar is conclusive, but an immature third molar is argumentative^45^. This perception is confirmed by our 3D CBCT data.

## Conclusion

Age estimation conducted by the rules of Demirjian and Nolla’s method may detect more advanced stages in minors. Nolla’s and Demirjian’s 2D and 3D imaging present significantly different staging results. Adulthood can only be safely assumed if the third molar is fully matured. In contrary, a vast number of individuals above the age of 18 present immature third molars.

## Methods

### Study design

The design of this study was a retrospective cross-sectional study of conventional panoramic radiographs and cone beam computer-tomography images of 312 patients aged 13–21 years who were treated in a single University Hospital for either dislocated lower jaw fractures or orthognathic surgery between January 2002 and April 2018.

### Sample

312 radiographic images of European patients were analyzed. The study’s cohort contained 127 females (40.7%) and 150 males (59.3%). The cohorts mean age was 16.2 (± 2.1 SD) years. The median was at 17 years. The chronological age for each patient was calculated by subtracting the date of the radiograph diagnostic from the date of birth after having converted both to a decimal age.

4 age groups were defined:

- Earlier adolescence (EA)—13 to 14 years of age.
- Adolescence (A)—15 to 16 years of age.
- Late adolescence (LA)—17 to 18 years of age.
- Young adults (YA)—19 to 21 years of age.

### Applied staging techniques for dental age determination

The developmental stages of the third molars were assessed according to the principles determined by Nolla^18^ and Demirjian^13^. In Nolla’s publication the age was determined by completion of calcification of permanent teeth which is divided into the stages 0–10^18^. In difference to Nolla’s publication, we applied the rules set by Nolla^18^ on the development of the third molar to investigated patient’s dental age. Likewise, we applied the method of dental age determination published by Demirjian^13^ for the permanent teeth of the lower left jaw (the first incisor to the second molar) on the third molar. In difference to Demirjian’s publication^13^, we labeled the stages numbers 1–8.

All staging techniques were solely applied in assessment of the lower jaw’s right third molars to maintain uniformity of the data. Two examiners (M.Zk. and L.B.) observed the radiographic images after a period of mutual calibration. To test intra- and inter-examiner reliability, two different examiners staged the development of third molars randomly selected radiographs. Each examiner repeated the process after 4 weeks (intra-examiner), and data from each examiner were compared (inter-examiner) to assess reliability. Two further examiners (J.B. and M.Z.) refereed the results.

### Statistics

Data were analyzed using SPSS statistical software (SPSS Version 24.0; IBM, Munich, Germany). For interval-scaled parameters the Bravais–Pearson coefficient was determined for the 2‐tailed correlation. Continuous variables were compared with the non-parametric Mann–Whitney-U, Wilcoxon signed-rank test or Kruskal–Wallis test, as appropriate. The Bonferroni adjustment was used to counteract the problem of multiple comparisons. The level of significance for p-values was set < 0.05. Descriptive analysis was performed as well.

### Ethical approval

All procedures performed in studies involving human participants were in accordance with the ethical standards of the institutional and/or national research committee and with the 1964 Helsinki declaration and its later amendments or comparable ethical standards. This study was approved by the local University Clinic of Cologne Germany Ethnic Committee (No.: 19-1525).

### Informed consent

Informed consent was obtained from parents/legally authorized representatives in case of minor patients (below 18 years of age) and informed consent was obtained from all other adult patients included in the study.
